# The Best Form of Medical Service

**Published:** 1921-02-05

**Authors:** Herbert Walters


### THE EDITOR'S LETTER-BOX.

**Correspondence on all subjects is invited, but we cannot in any way be responsible for the opinions expressed by our correspondents, who must give their name and address as a guarantee of good faith, but not necessarily for publication. Correspondents are reminded that brevity of style and conciseness of statement greatly facilitate early insertion.**

### THE BEST FORM OF MEDICAL SERVICE.

*To the Editor of* The Hospital.

Sir,—I was much impressed with the leading article in your issue on January 22, mainly by reason of the soundness of your advice that the public should not accept or be led by opinions or statements in the Press merely on account of their constant reiteration, but should take a share in bringing to light by means of discussion the maximum amount of data.

In the world of hospital affairs we have seen during the past year or two the enormous damage done to our credit and to our fund-raising departments by the repetition of the statement that the State is about to take over the hospitals, and even to-day, when appealing for funds, we are met with this argument, in spite of the definite statements made in the House of Commons and outside that the Government do not propose to adopt this course.

I hesitate to take up your valuable space to deal with any but the hospital aspects of the problem of State service, but I feel it necessary to educe the following arguments on the main principles in order to make the hospital position clear.

It ought not to be necessary to emphasise the benefits arising from life, endowment, educational, fire, marine, or any other kind of insurance, and the principle of insuring for medical attendance must surely be sound. Such insurance under a voluntary system becomes a difficult actuarial matter, and its benefits to the general community may not accrue for ten or twenty years, but that does not detract from its merits.

On the other hand, enforced insurance, with the information now at our command, is a simpler problem and its benefits are immediate. Possibly a combination of the two would be found of the greatest use, that is to say, compulsory insurance for those in receipt of, say, up to £250 per annum, and a voluntary scheme offered to those above this limit.

So far as it concerns the medical profession, one must confess from experience of the present Act that State medicine has not so far tended to good medicine. The amount of clerical work inseparable from control detracts either in quantity or quality from the medical service which the practitioner can render; it must have the tendency of disturbing the personal equation between patient and medical man and to remove the medical man from the position of being a family friend and adviser. This must have its effect upon the patient; but, what is far more important from the patient's point of view, under an insurance system the patient is tied to one practitioner for a specified period of three or six months, or whatever may be laid down, whereas the friends, distraught with anxiety, may be in immediate need of a change of practitioner, but are precluded from taking action without a period of notice.

The administration of the finance of the present system leaves much to be desired. It is obviously bad finance to appropriate balances accruing in respect of patients who have not chosen their practitioners and to distribute them amongst the practitioners on the panel. It is unworthy of a big spending department of the Government to pay amounts to practitioners for patients who remain upon their panel but who never consult them and go elsewhere when in need of advice. It is against the patient's interests for the panel practitioner's pharmacopœia to be unduly limited.

It follows that many of these actions will have their reactions upon the hospitals.

Ten years or more ago when the Insurance Act was under consideration the policy of the hospitals became settled when it was found that the Insurance Act gave to insurance patients the benefits of a general practitioner's advice and treatment and no more.

Sir Napier Burnett stated at a meeting a few weeks ago that there is a large balance, amounting to something like fifty million pounds, unappropriated on account of the National Insurance Act, and that the hospitals should have a reasonable claim to receive some of this money.

Before this could be done I venture to submit that an amending Act would be necessary. I should strongly support such an extension of the Insurance Act, and I consider that a State medical service should similarly contain provisions for hospital treatment.

At the present moment the insured person is considered eligible for both in-patient and out-patient hospital treatment. Should the scope of National Insurance be widened into a State medical service without hospital treatment it is quite apparent that a large number of insured people would not be eligible as out-patients and a smaller number would be ineligible as in-patients. The position of the hospitals in regard to patients contracted for under such a service would require very earnest consideration.

-Believe me, yours faithfully.

Herbert Walters.

